# Expression of pim-1 in Tumors, Tumor Stroma and Tumor-Adjacent Mucosa Co-Determines the Prognosis of Colon Cancer Patients

**DOI:** 10.1371/journal.pone.0076693

**Published:** 2013-10-07

**Authors:** Yong-hai Peng, Jian-jun Li, Fang-wei Xie, Jian-fang Chen, Ying-hao Yu, Xue-nong Ouyang, Hou-jie Liang

**Affiliations:** 1 Department of Oncology, Southwest Hospital, Third Military Medical University, Chongqing, People’s Republic of China; 2 Department of Oncology, Fuzhou General Hospital, Fuzhou, People’s Republic of China; 3 Department of Pathology, Fuzhou General Hospital, Fuzhou, People’s Republic of China; Kyushu University Faculty of Medical Science, Japan

## Abstract

Provirus integration site for Moloney murine leukemia virus (pim-1) is a proto-oncogene that is linked to the development and progression of several cancers. In this study, we evaluated pim-1 expression in tumors, tumor stroma and tumor-adjacent mucosa together as an independent prognostic factor for colon cancer patients. The study included 343 colon cancer patients. Immunohistochemical staining was used to detect pim-1. Multivariate cox regression for disease-free survival (DFS) were used to identify independent prognostic factors. Analytic hierarchy process (AHP) was used to calculate the weight of pim-1 in tumors, tumor stroma and tumor-adjacent mucosa in order to obtain a Pim-1 total score (PTS) for recurrence and survival. Kaplan–Meier DFS curves and OS curves for patients with different pim-1 expression levels were compared using the log-rank test. In this study, four independent prognostic factors were identified for colon cancer patients: pim-1 expression in tumors, tumor stroma, tumor-adjacent mucosa, as well as tumor stage. It has been established that clinical stage is an important prognostic factor for colon cancer patients. However, PTS can identify the patients who are likely to recur not only in the whole radical excision group but also within each stage of this group. Based on the results of this study we can conclude that the PTS combined with clinical staging system may be a better predictor of colon cancer patients’ prognosis than using the clinical stage system alone.

**Clinical Trials Gov. Number:** ChiCTR-PRCH-12002842

## Introduction

Recurrence and distal metastasis are the main causes of death in patients with colon cancer. The currently available staging system is unable to discriminate between those patients who will recur and die and those patients who will remain disease-free because the prognosis of colon cancer patients is determined not only by the characteristics of the tumor cell itself but also by the cancerous status of the adjacent mucosa [1], the activation of immune cells and the presence of other tumor stroma components [2,3]. Thus, the identification and evaluation of new prognostic biomarkers is crucial in order to identify high-risk patients and to ensure that these patients receive the necessary treatment.

The proto-oncogene Provirus integration site for Moloney murine leukemia virus (PIM1) encodes a serine/threonine protein kinase which regulates cell survival, proliferation, differentiation, apoptosis and tumorigenesis [4]. The Pim family has three members: pim-1, pim-2 and pim-3. Of them all, pim-1 is considered to be the most useful biomarker for cancer diagnosis and prognosis [5,9]. The Pim-1 gene encodes 2 isoforms with molecular weights of 33 and 44 kDa, respectively [6]. The 44 kDa PIM-1 mainly localizes to the membrane, whereas the 33 kDa pim-1 localizes to the cytosol and nucleus, indicating that these two isoforms regulate distinct signaling pathways in cancer cells [7,8]. Recent studies of pim-1 have focused on its oncogenic activities, and possible use of Pim kinases inhibition as a target for therapeutic intervention [9]. Overexpression of Pim-1 kinases was observed in human tumors of hematopoietic and epithelial origin. In addition, it correlated with poor prognosis in several hematopoietic malignancies [10,11], gastric cancer [12] and squamous cell carcinoma of the head and neck [13]. Nevertheless, contrary to these findings, Pim-1 overexpression correlated with good prognosis in prostate cancer, pancreatic ductal carcinoma and non-small cell lung cancer [14-17].

During embryonic development, PIM-1 is highly expressed in liver, spleen, bone marrow, while its expression is absent in adult tissues [18]. Other studies of the physiological activities of Pim-1 kinases revealed that pim1-deficient mice failed to support the growth of pre-B cells [19]. Moreso, Pim1-deficient hematopoietic stem cells could not re-populate irradiated hosts [20]. In addition, other studies revealed that CD40, a TNFR family member that plays a central role in T cell-dependent B cell activation, up-regulates the expression of pim-1 to maintain B cell survival and proliferation [21]. Organisms lacking CD40 or CD40L are unable to generate secondary humoral immune responses to T cell-dependent antigens [22]. Thus, pim-1 expression in tumor stroma may be an important biomarker reflecting the ability of T or B cells to produce an immune response against the tumor.

The known functions of pim-1 suggest that it may possibly serve as a good marker to evaluate tumor properties, immune activation of tumor interstitial leukocytes, and the cancerous status of the tumor-adjacent mucosa. In this study, we used a tissue microarray (TMA) database to define combinations of tumor, tumor stroma and tumor-adjacent mucosa associated with DFS and OS in colon cancer. In conjunction with the current clinical staging system, the information obtained from the database improved our ability to identify patients at high risk for disease recurrence after radical excision.

## Methods

### Clinical data and colon cancer sample collection

This study was approved by the Ethics Committee of Southwest Hospital and Fuzhou General Hospital. Patients provided written informed consent according to GCP that their specimens will be used as part of the ongoing study on molecular genetics of colon cancer. In this study, clinical samples were taken from patients with colon cancer that underwent radical colectomy, between 2004 and 2010 at Fuzhou General Hospital. Colectomy specimens from 343 patients were used to construct the TMAs. All cases had sufficient cancer tissue available at colectomy for representative cancer tissue to be harvested for TMA construction after the standard clinical histopathologic diagnosis. Clinical post-colectomy follow-up data, including regular patient assessment by clinic visit, phone, mail contact or community service station, were used to ascertain overall cancer-specific and disease-free survival. The focal recurrence was determined by colonoscopy and the distant metastases were determined by CT scan. All patients were properly advised to undergo necessary therapy and health examination according to the National Comprehensive Cancer Network (NCCN) guidelines. In this study, the closing date for follow-up was December, 2011.

### TMA construction

High-density tissue microarrays were constructed as described by Perrone et al. [23] TMAs were assembled with the TM-1 tissue arrayer (Beijing boyikang Laboratory Instrument Co., Ltd., China). Normal blank paraffin blocks used in clinical diagnosis were used for the recipient array blocks. All clinical hematoxylin and eosin (H&E)-stained slides were examined by histology to verify the targeted tissues. The mucosa present within l cm of the tumor mass was defined as the tumor-adjacent mucosa. Up to two replicate tissue cores were taken from each circled area. To construct a TMA, core centers from tissue cores with a 1.2 mm diameter were spaced 1.6 mm apart. Then, the TMA was thawed in an incubator for 1 h to a semi-liquid state at 60°C. After the TMA construction, 4µm sections were cut, and the initial slide was stained with H&E to verify the histologic diagnosis. The TMA images were acquired with the BLISS microscope imaging workstation (Bacus Laboratories, Inc., Lombard, USA). Protein expression was evaluated with an image pro-plus 6.0 software (Media Cybernetics, USA) by two senior pathologists independently.

### Immunohistochemistry

Standard biotin-avidin peroxidase-complex immunohistochemistry was used to analyze the pim-1 protein expression. The rabbit monoclonal antibody against PIM1 (Epitomics, catalog＃2409-1) at a dilution of 1:150 (original concentration 0.2 mg ml^-1^) was used. The expression level of pim-1 was denoted as absent, weak, moderate or strong according to the immunostaining intensity and the percent of positive cells. The staining intensity was evaluated in a blinded fashion and graded on a scale of 0 (negative) to 2+ (strong positive), as reported [8]. The extent of positive staining in the tumor-adjacent mucosa and tumor foci was classified as 0,1-25 (1+), 26-50 (2+), 50-75 (3+) and 76-100% (4+) as reported [8]. Absent (0) and weak level (1+) pim-1 expression was scored as 1 (low), moderate (2+) as 2 (moderate) and high level (3+, 4+) pim-1 expression was scored as 3 (high). The scoring was performed by two senior pathologists independently using a telepathology system without prior knowledge of stage or clinical outcome. A total of 42 tissue microarray blocks from 540 colon cancer samples derived from 343 patients were assessed. A median score was calculated from two colon cancer samples taken from each patient.

### Statistical methods

Cox regression was used to evaluate whether sex, age, tumor size, lymph node metastasis, remote organ metastasis, clinical stage, degree of differentiation and pim-1 expression levels in colon cancer samples (which included tumors, tumor stroma and tumor-adjacent mucosa) are in correlation with DFS and OS. The Delphi Method was employed to obtain the intensities for the AHP. Then AHP was used to calculate the weights of pim-1 contributing to DFS in tumors, tumor stroma and tumor-adjacent mucosa in order to obtain the pim-1 total score (PTS) (Table 1). Then PTSs were classified into low, moderate and high grades by the Delphi Method. Kaplan-Meier analysis was used to estimate the cumulative percentage of DFS and OS based on different tumor stages, pim-1 expression level in tumors, tumor stroma and tumor-adjacent mucosa as well as PTS, and log-rank test was used to compare the Kaplan-Meier curves.

**Table 1:** To obtain a pim-1 total score (PTS), the weight of pim-1 in tumors, tumor stroma and tumor-adjacent mucosa was caculaed by analytic hierarchy process.

PIM-1 expression	Absent or weak	moderate	high	Mi	Wi	Wi0	characteristic vector	maximum eigenvalue	Consistency Index (CI)
tumor	1	1/3	1/5	0.066666667	0.40548013	0.1132686	0.35829525	3.16323453	0.081617266
Tumor-adjacent mucosa	3	1	2	6	1.81712059	0.5076026	1.60566604		
Tumor stroma	5	1/2	1	2.5	1.35720881	0.3791288	1.19927324		
					3.57980953				

## Results

### Baseline characteristics of clinical patients

In order to determine whether the dataset was representative, the characteristics of the cohort of recruited patients were first examined. A total of 343 patients were recruited for this study and the patients’ baseline characteristics are presented in Table 2. The average age of patients at surgery was 61 years (range 17–86 years). Clinical stage was determined upon post-operation pathological examination in accordance with the AJCC Cancer Staging Manual (7th ed). There were 52, 186 and 106 patients in stages I, II and III, respectively. One patient was rejected because of R1 excision in stageⅡdesease. The mean DFS time was 61 months (range 0.5–86 months).

**Table 2:** Clinicopathologic correlation of pim-1 expression in colon cancer.

	Pim-1 expression in tumor (%) Pim-1 total score (PTS) (%)											
Variables	low		moderate		high	*P ^※^*	Low	moderate		high		*p* ^※^
**Age (years)**						0.6465						0.2424
<60 y	18.79		41.82		39.39		27.88		46.67		25.45	
	(31/165)		(69/165)		(65/165)		(46/165)		(77/165)		(42/165)	
≥60 y	16.29		46.63		30.08		30.34		51.69		17.98	
	(29/178)		(83/178)		(66/178)		(54/178)		(92/178)		(32/178)	
**Gender**						0.6300						0.1464
Male	16.33		46.43		37.24		33.16		47.45		19.39	
	(32/196)		(91/196)		(73/196)		(65/196)		(93/196)		(38/196)	
Female	19.05		41.50		39.46		23.81		51.70		24.49	
	(28/147)		(61/147)		(58/147)		(35/147)		(76/147)		(36/147)	
**WHO grade**						0.7067						0.2357
G1	20.41	36.73		42.86			28.57		48.98		22.45	
	(10/49)	(18/49)		(21/49)			(14/49)		(24/49)		(11/49)	
G2	17.62	44.83		37.55			30.65		49.81		19.54	
	(46/261)	(117/261)		(98/261)			(80/261)		(130/261)		(51/261)	
G3	12.12	51.51		36.36			18.18		45.45		36.36	
	(4/33)	(17/33)		(12/33)			(6/33)		(15/33)		(12/33)	
**T status**						0.5979						0.3901
T 0/1	16.67		16.67		66.67		50.00		16,67		33.33	
	(1/6)		(1/6)		(4/6)		(3/6)		(1/6)		(2/6)	
T2	18.87		37.74		43.40		22.64		56.60		20.75	
	(10/53)		(20/53)		(23/53)		(12/53)		(30/53)		(11/53)	
T3	17.92		45.00		37.08		31.67		47.50		20.83	
	(43/240)		(108/240)		(89/240)		(76/240)		(114/240)		(50/240)	
T4	13.64		52.27		34.09		20.45		54.55		25.00	
	(6/44)		(23/44)		(15/44)		(9/44)		(24/44)		(11/44)	
**N status**						0.7581						0.0330
N0	16.88		44.73		38.40		32.49		49.79		17.72	
	(40/237)		(106/237)		(91/237)		(77/237)		(118/237)		(42/237)	
N1	22.58		43.55		33.87		29.03		46.77		24.19	
	(14/62)		(27/62)		(21/62)		(18/62)		(29/62)		(15/62)	
N2	17.39		34.78		47.83		8.70		52.17		39.13	
	(4/23)		(8/23)		(11/23)		(2/23)		(12/23)		(9/23)	
N3	9.52		52.38		38.10		14.29		47.62		38.10	
	(2/21)		(11/21)		(8/21)		(3/21)		(10/21)		(8/21)	
**TNM stages**						0.7329						0.0510
Stage i	19.23		36.54		44.23		26.92	55.77		17.31		
	(10/52)		(19/52)		(23/52)		(14/52)	(29/52)		(9/52)		
Stage ii	16.22		47.03		36.76		34.05	48.11		17.84		
	(30/185)		(87/185)		(68/185)		(63/185)	(89/185)		(33/185)		
Stage iii	18.87		43.40		37.74		21.70	48.11		30.19		
	(20/106)		(46/106)		(40/106)		(23/106)	(51/106)		(32/106)		

※ Chi-square test. Abbreviation: T, tumor; N, node.

### The relationship between Pim-1 expression and DFS or OS

**Figure 1:**
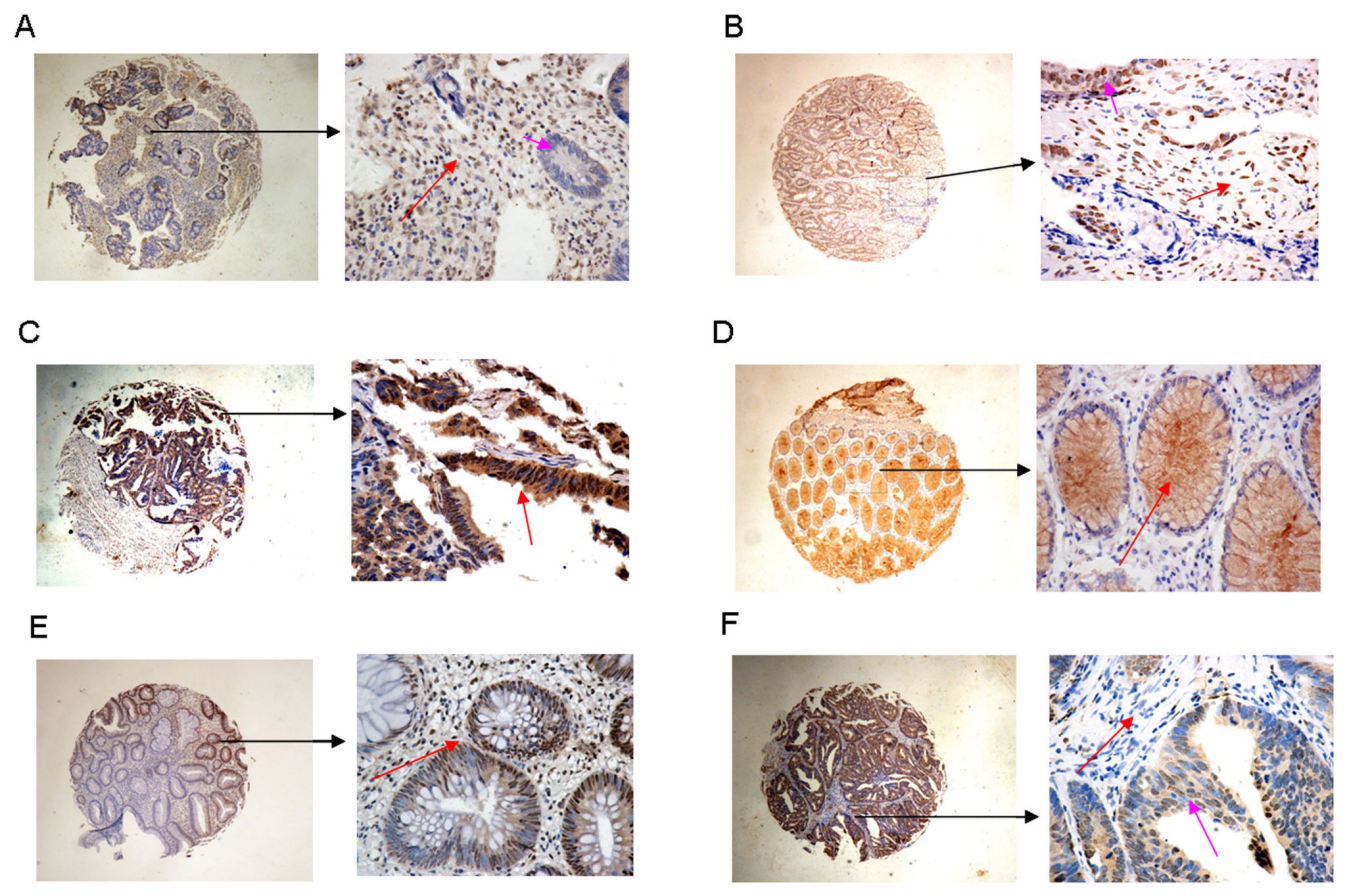
The profile of Pim-1 expression in colon cancer. (A) High level of Pim-1 expression in tumor stroma (red arrow), score 3, low level of pim-1 expression in tumor (pink arrow), score 1. (B) High level of pim-1 expression in tumor stroma (red arrow), score 3, high level of Pim-1 expression in tumor (pink arrow), score 3. (C) High level Pim-1 expression in tumor (red arrow), score 3. (D) High level of Pim-1 expression in tumor-adjacent mucosa (red arrow), score 3. (E) pim-1 in clusters within the nuclei of tumor-adjacent mucosa cells scored as fairly strong (red arrow), score 3. (F) Low level of Pim-1 expression in tumor stroma (red arrow), score 1, median level expression of pim-1 in tumor (pink arrow), score 2.

Pim-1 is usually localized on the membrane, cytosol and nucleus of tumor cells, tumor stroma cells as well as tumor-adjacent mucosa cells, as previously reported [8]. The different scores of pim-1 expression are presented in Figure 1 and Figure S1. We must note that the location of pim-1 in clusters within the nuclei of tumor-adjacent mucosa cells was scored as fairly strong (Figure 1E). Low, medium and high Pim-1 expression was observed in 17.49% (60 of 343), 44.31% (152 of 343) and 38.19% (131 of 343) of patient tumors, respectively (Table 2). When tumor stroma was examined 34.99% (120 of 343), 44.61% (153 of 343) and 20.41% (70 of 343) of patients exhibited low, medium, and high expression of Pim-1 (Table S1), respectively. In tumor-adjacent mucosa, 63.56% (218 of 343), 29.15% (100 of 343) and 7.29% (25 of 343) of patients exhibited low, medium, and high Pim-1 expression (Table S1), respectively.

In a multivariable Cox proportional hazards model, tumor status (P=0.015), TNM stage (P=0.000), pim-1 expression in tumor stroma (P=0.000) and pim-1 expression in tumor-adjacent mucosa (P=0.000) were each independently and significantly associated with DFS (Table 3). In addition, pim-1 expression in tumor stroma significantly associated with DFS.

**Table 3:** Multivariate Cox regression analysis for DFS in 343 patients with colon cancer.

	DFS (n=343)	
	95.0% CI for Exp(B)	*P* value
T status	1.558 (1.090,2.228)	0.015
TNM Stages	1.910 (1.449,2.516)	0.000
Pim-1 in Tumor stroma	0.387 (0.286,0.525)	0.000
Tumor adjacent mucosa	4.463 (3.261,6.107)	0.000

**Figure 2:**
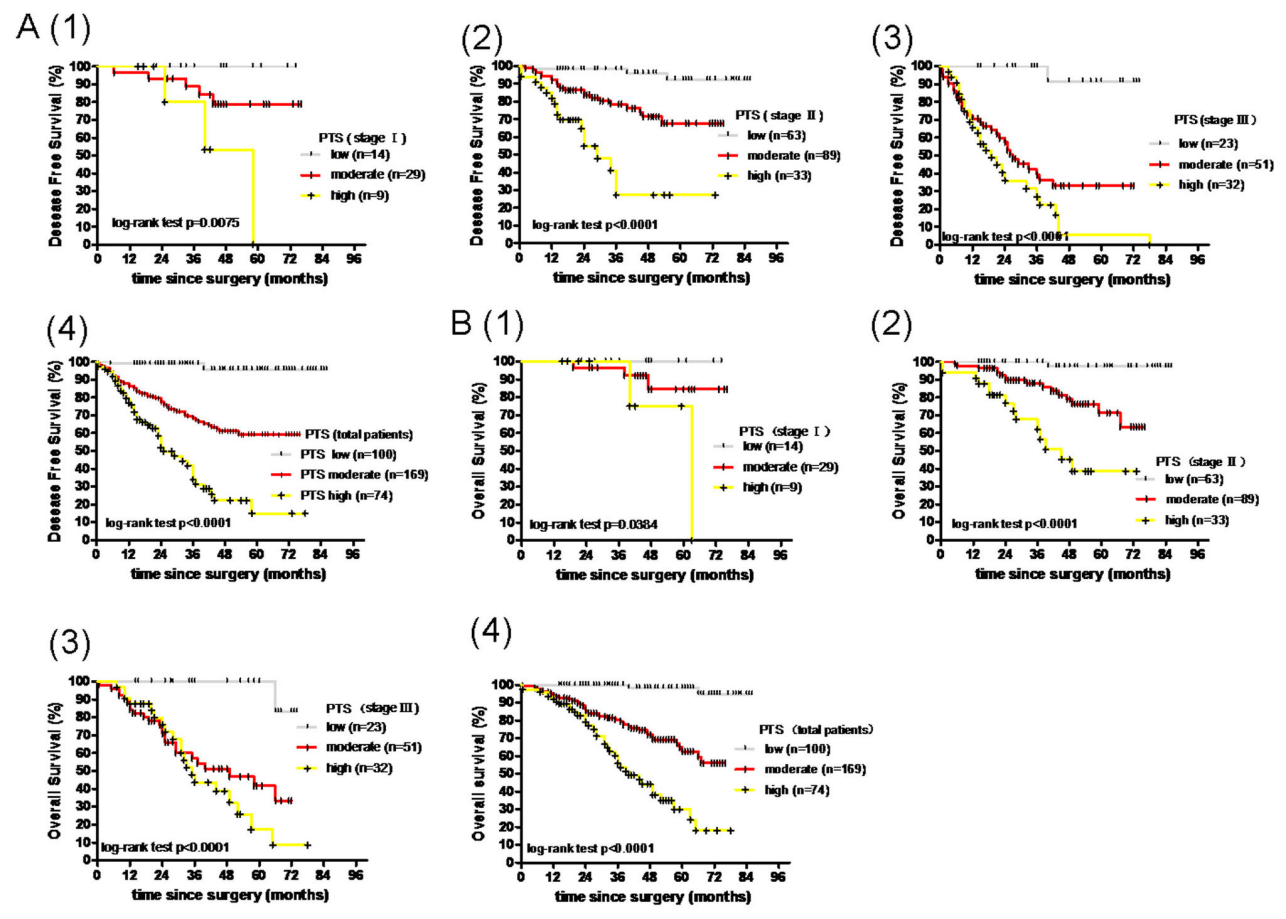
Survival curves of colon cancer patients generated by Kaplan–Meier analyses. (A) DFS curves of different PTS (pim-1 total score) in different groups of patients: 1, stage Ⅰ; 2, stage Ⅱ; 3, stage Ⅲ; 4, all patients. (B) OS curves of different PTS (pim-1 total score) in different groups of patients: 1, stage Ⅰ; 2, stage Ⅱ; 3, stage Ⅲ; 4, all patients.

These combined characteristics of pim-1 expression in tumors, tumor stroma and tumor-adjacent mucosa suggest that pim-1 expression might serve as a useful prognostic marker for colon cancer. To test this hypothesis, we used analytic hierarchy process (AHP) to determine the weighted coefficient distribution of pim-1 contributing to DFS and OS in tumors, tumor stroma and tumor-adjacent mucosa which was 0.1132686, 0.3791288 and 0.5076026, respectively (Table 1). Next, the PTS was calculated based on the pathological score, using the formula PTS=0.1132686p1+0.5076026p2+ (-)0.3791288p3, where p1, p2 and p3 corresponded to the pathological score of the pim-1 expression in tumor, tumor-adjacent mucosa and tumor stroma, respectively. PTS were further classified into low (PTS≤0), moderate (0<PTS≤0.5) and high (PTS >0.5) grades by Delphi Method. Using the Kaplan–Meier method with the log-rank test, DFS curves (Figure 2.A.4) and OS curves (Figure 2.B.4) were compared. These analyses have further demonstrated that the PTS evaluation combined with clinical stage is more accurate than evaluation by clinical stage alone (Figure 2.A,B).

### The relationship between Pim-1 expression and DFS or OS in different tumor stages

Patient survival was analyzed by the Kaplan–Meier, log-rank test (Figure S2.D.1; Figure S3.D.1), as were the DFS and OS based on clinical stage (Figure S2.D.2; Figure S3.D.2; Table 4, S2-S4). The results of these analyses indicate that the pim-1 expression in tumor-adjacent mucosa and tumor stroma, as well as PTS were significantly associated with DFS (P<0.0001, P=0.0041, P< 0.0001, respectively) and OS (P<0.0001, P=0.0066, P< 0.0001, respectively) (Table 4, Figures 2.A.4 and B.4; Figures S2.B.4 and C.4; Figures S3.B.4 and C.4).

**Table 4:** Predictive variables for DFS and OS of 343 patients with colon cancer by univariate survival analysis.

Variables	P cases	DFS		OS	
		5 years’ survival,%	p^※^	five years survival,%	p^※^
**Pim-1 (tumors)**	343		0.9096		0.9126
low	60	52.96		65.69	
moderate	152	63.88		65.69	
high	131	62.60		68.93	
**pim-1 (tumor-adjacent mucosa)**	343		<0.0001		<0.0001
low	218	80.59		84.49	
moderate	100	36.28		40.27	
high	25	0		0	
**pim-1 (tumor stroma)**	343		0.0041		0.0066
low	120	30.23		34.32	
moderate	153	70.69		78.02	
high	70	68.85		69.34	
**PTS**	343		<0.0001		<0.0001
low	100	95.75		98.36	
moderate	169	59.29		62.57	
high	74	14.92		30.03	
**TNM stages**	343		<0.0001		<0.0001
Stage Ⅰ	52	75.65		81.09	
Stage Ⅱ	185	72.54		75.81	
Stage Ⅲ	106	22.31		46.30	

※ log-rank test.

Furthermore, we have examined the DFS and OS in each tumor stage based on pim-1 expression in tumors (Figure S2. A.1,2,3,4; Figure S3. A.1,2,3,4), tumor-adjacent mucosa (Figure S2. B. 1,2,3,4; Figure S3. B.1,2,3,4), tumor stroma (Figure S2. Cs. 1, 2, 3, 4; Figure S3. Cs. 1, 2, 3, 4), as well as PTS (Figure 2. A. 1,2,3,4 and B. 1,2,3,4). We found that PTS not only can predict the DFS but also the OS of colon cancer patients (Table S2-S4, Figure 2.A.1,2,3 and B. 1,2,3).

To test the hypothesis that the cancer status of the adjacent mucosa and the activation of immune cells in tumor stroma were more important prognostic factor in the disease-free period, while the tumor characters are more important prognostic factor in the disease relapse/metastasis period, we have examined the relationship between progression-free survival (PFS) and the pim-1 expression in tumor, tumor-adjacent mucosa and tumor stroma. Fifty two patients who received first line chemotherapy with 5-FU plus Oxaliplatin were recruited for this purpose, after recurrence. The results of these analyses show that the PFS was significantly associated with pim-1 expression in tumor (p= 0.0141) while there was no statistically significant association between PFS and pim-1 expression in tumor-adjacent mucosa (p=0.6194) or tumor stroma (p=0.3438) (data not shown). These results indicate that PTS can reflect the cancer status of the adjacent mucosa and the activation of immune cells in tumor stroma as well as tumor characteristics. In short, PTS score can improve prediction beyond clinical stage than any other significant predictor for DFS.

## Discussion

The incidence of colon cancer is still increasing, especially in the developed countries [24]. Surgical resection of the primary tumor is the primary treatment for colorectal cancer with the exception of stage Ⅰ and in part stage II colon cancers for which the administration of adjuvant chemotherapy is recommended by NCCN GUIDELINE (http://www.nccn.org/index.asp).

The use of adjuvant chemotherapy to treat stage II colon cancer is still under dispute and even in stage III patients the adherence to the NCCN Guidelines is 71% [25]. The American Society of Clinical Oncology reports that the evidence collected from the randomized controlled trials does not support the use of adjuvant chemotherapy as the routine treatment for patients with stage II colon cancer, however the patients with high-risk stage II disease may benefit from this kind of treatment [26]. In addition the high-risk patients are not well defined. The results of the MOSAIC trial indicated that adding the Oxaliplatin to a fluorouracil and leucovorin regimen improves the adjuvant treatment of colon cancer (stage II and Ⅲ) [27]. However, the benefit from the adjuvant treatment often comes at the cost of many patients’ receiving excessive treatment even in stage Ⅲ of the disease.

Today, tumor stage is still the most important prognostic indicator used in the clinic to predict survival of patients with early-stage colon cancer. Nevertheless, the accurate prediction of the colon cancer recurrence is still not achieved and there is a persistent need for better markers in order to resolve this problem. Recent studies aimed at identifying other prognostic factors have mainly been focused on examining tumor cells, as it was the case in studies examining the importance of miR-365 [28], intestinal stem cell signature [29], thymidilate synthase [30], and other possible candidate biomarkers. Each of these studies is limited to theoretical examinations, which are difficult to put into clinical practice. In this study, we have developed a method combined with clinical stages that can predict the recurrence of colon cancer in patients with radical colectomy.

Although there was no direct evidence that they should receive adjuvant chemotherapy in stage Ⅱ disease, there were still 42% of patients included in our study who have received 5-FU adjuvant chemotherapy, 12% who have received capecitabine and 15% of patients who received 5-FU plus Oxaliplatin adjuvant chemotherapy while the remaining 31% patients did not receive adjuvant chemotherapy. The main reason why they received adjuvant chemotherapy was the doctor-patient psychology comforts.

In addition, our study included 42% patients who received 5-FU plus oxaliplatin adjuvant chemotherapy, 39% patients who received 5-FU alone or capecitabine alone adjuvant chemotherapy, and 19% patients who refused to receive chemotherapy in stage III disease. There was no significant difference (p>0.05) in DFS or OS between stage II patients regardless of whether they received adjuvant chemotherapy or not. In addition, there was no significant difference (p>0.05) between different subgroups of stage III patients again regardless of whether they received adjuvant chemotherapy or not. Furthermore in order to avoid the effect of subsequent treatment after recurrence, in this study, we took DFS as the primary endpoint, and OS as the secondary endpoint.

Intestinal stem cells may be the cells of origin for colorectal cancer [31]. Given that the oncogenic activation of intracellular signaling pathways has been associated with tumorigenesis in colon cancer cells [32], oncogene activation of tumor-adjacent mucosa might possibly be used in prediction of tumor recurrence after radical colectomy. Our data demonstrate that pim-1 oncogene activation of tumor-adjacent mucosa is significantly associated with DFS and that higher level of pim-1 expression account for lower DFS. Therefore oncogene activation in the tumor-adjacent mucosa should be taken into account when studies are conducted to identify prognostic factors in colon cancer. However these results are contradictory to clinical findings that the recurrence of the disease after colectomy is mainly due to the remote metastasis and not focal recurrence and therefore further studies are warranted.

In the last decade, cancer-associated stroma has become a greater concern than insular masses [33-35]. Cancer-associated fibroblasts are the main component of cancer-associated stroma. The significance of different subtypes of cancer-associated fibroblasts in tumor pathogenesis is not clear. In our study, fibroblasts were found in various proportions in colon cancers, often constituting the main population of cells in the tumor stroma. Interestingly, in some cases, cancer-associated fibroblasts were positive for pim-1 expression, whereas in other cases they were pim-1 negative. This phenomenon caught our interest, and we have found that higher levels of pim-1 expression in cancer-associated stroma were associated with higher DFS and OS. Our data further indicated that pim-1-positive cancer-associated tumor stroma cells were protective to the host, but the exact mechanism of their action still remains to be elucidated.

Inflammation has a well-established role in promoting tumor progression [36]. The inflammatory microenvironment is an essential component of all tumors. Thus, immune inflammatory cells are an additional component of cancer-associated stroma. Most studies have focused on the ability of inflammatory cells to promote tumorigenesis, development, invasion and metastasis [2,37]. Moreover, a recent study found that increased infiltration of tumors with cytotoxic CD8-positive T cells has been correlated with prolonged survival [38].

In our study of colon cancer, we found that, in contrast to pim-1-negative immune inflammatory cells, pim-1-positive immune inflammatory cells that accompany cancer-associated fibroblasts were associated with increased DFS and OS, perhaps acting as tumor-killing subclasses of immune cells. This result indicates that pim-1 can act as a potential marker to discriminate between tumor-promoting and tumor-killing immune inflammatory cells which is consistent with previous studies [39].

In conclusion, our study suggests that the prognostic parameters of colon cancer are not only determined by tumor cells but also by cancer-associated stroma and tumor-adjacent mucosa especially when it comes to DFS. Therefore the activation of the proto-oncogene pim-1 in different parts of tumor (tumor cells, cancer-associated stroma and tumor-adjacent mucosa) may be very important for the evaluation of patient prognosis. Nevertheless further studies in order to elucidate the exact mechanism of pim-1 action in tumors and tumor associated mucosa, are still warranted.

## Supporting Information

Figure S1 **The profile of Pim-1 expression.** (A) Median level expression of Pim-1 in tumor stroma (red arrow), scores 2, high level expression of Pim-1 in tumor (pink arrow), scores 3. (B) Negative expression of Pim-1 in tumor stroma (red arrow), scores 1. (C) Negative expression of Pim-1 in tumor (red arrow), scores 1. (D) Median level expression of Pim-1 in tumor-adjacent mucosa (red arrow), scores 2.
(TIF)

Figure S2 **DFS curves generated by Kaplan–Meier analyses.** (A) DFS curves of different pim-1 expression level in tumor of different group patients: 1, stage Ⅰ; 2, stageⅡ; 3, stage Ⅲ; 4, total patients. (B) DFS curves of different pim-1 expression level in tumor stroma of different group patients: 1, stage Ⅰ; 2, stageⅡ; 3, stage Ⅲ; 4, total patients. (C) DFS curves of different pim-1 expression level in tumor-adjacent mucosa of different group patients: 1, stage Ⅰ; 2, stageⅡ; 3, stage Ⅲ; 4, total patients. (D) DFS curves of total patients (1) and patients with stage Ⅰ, Ⅱ, Ⅲ diseases (2).
(TIF)

Figure S3 **OS curves generated by Kaplan–Meier analyses.** (A) OS curves of different pim-1 expression level in tumor of different group patients: 1, stage Ⅰ; 2, stageⅡ; 3, stage Ⅲ; 4, total patients. (B) OS curves of different pim-1 expression level in tumor stroma of different group patients: 1, stage Ⅰ; 2, stageⅡ; 3, stage Ⅲ; 4, total patients. (C) OS curves of different pim-1 expression level in tumor-adjacent mucosa of different group patients: 1, stage Ⅰ; 2, stageⅡ; 3, stage Ⅲ; 4, total patients. (D) OS curves of total patients (1) and patients with stage Ⅰ, Ⅱ, Ⅲ diseases (2).
(TIF)

Table S1 **Clinicopathological correlation of pim-1 expression in colon cancer.**
(DOC)

Table S2 **Predictive variables for DFS and OS of patients with stage i disease by univariate survival analysis. (※log-rank test.).**
(DOC)

Table S3 **Predictive variables for DFS and OS of patients with stage ii disease by univariate survival analysis.**
(DOC)

Table S4 **Predictive variables for DFS and OS of patients with stage iii disease by univariate survival analysis.**
(DOC)

Table S5 **Pim-1 expression in tumors, tumor-adjacent mucosa and tumor stroma.**
(DOC)
