# Supplementary figures and images for: Expression of pim-1 in Tumors, Tumor Stroma and Tumor-Adjacent Mucosa Co-Determines the Prognosis of Colon Cancer Patients
Source: PLoS One. 2013 Oct 7;8(10):e76693. doi: 10.1371/journal.pone.0076693 (PMC3792018; doi:10.1371/journal.pone.0076693)

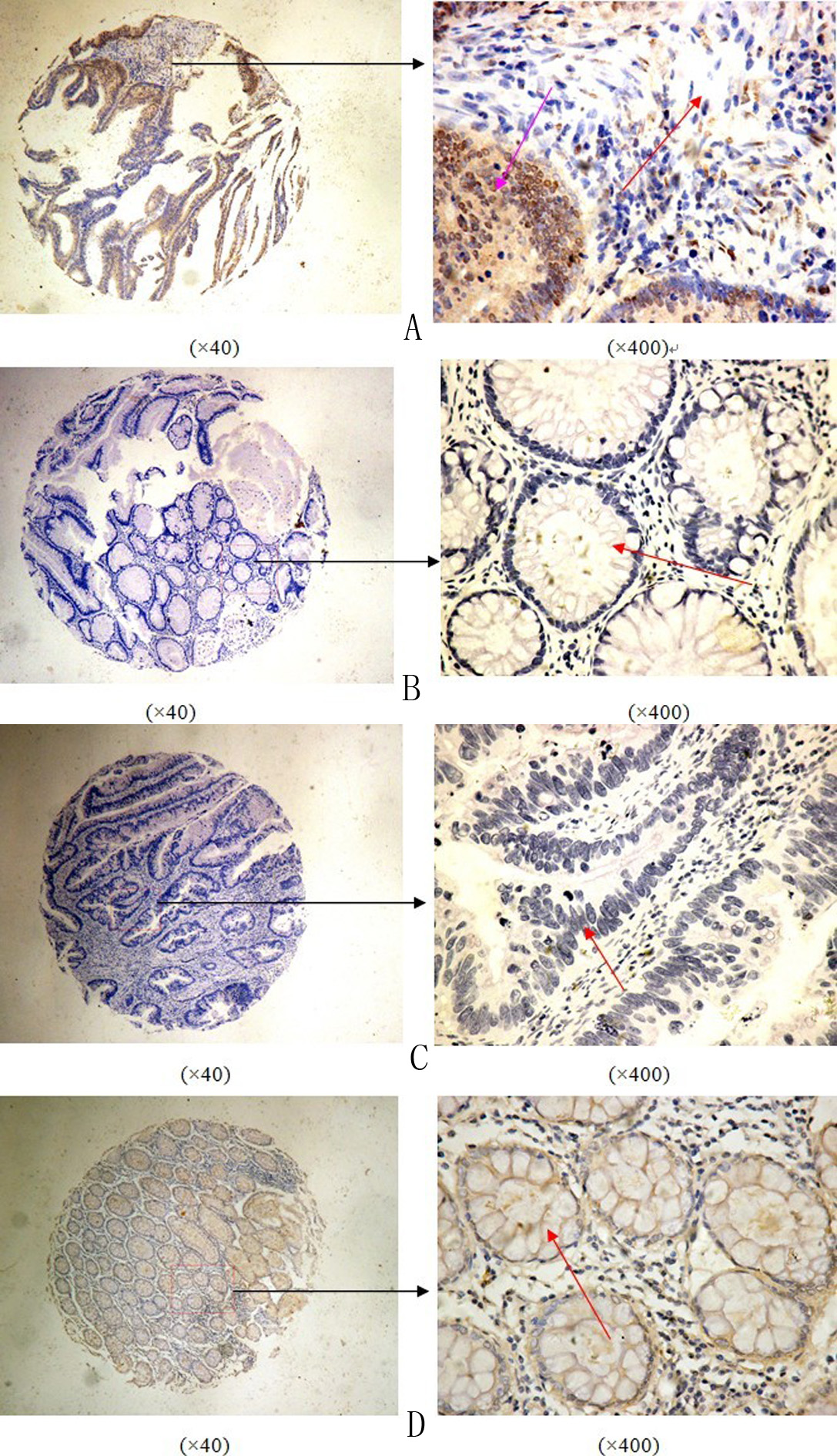

Supplement: Figure S1 — The profile of Pim-1 expression. (A) Median level expression of Pim-1 in tumor stroma (red arrow), scores 2, high level expression of Pim-1 in tumor (pink arrow), scores 3. (B) Negative expression of Pim-1 in tumor stroma (red arrow), scores 1. (C) Negative expression of Pim-1 in tumor (red arrow), scores 1. (D) Median level expression of Pim-1 in tumor-adjacent mucosa (red arrow), scores 2. (TIF) [file pone.0076693.s001.tif]

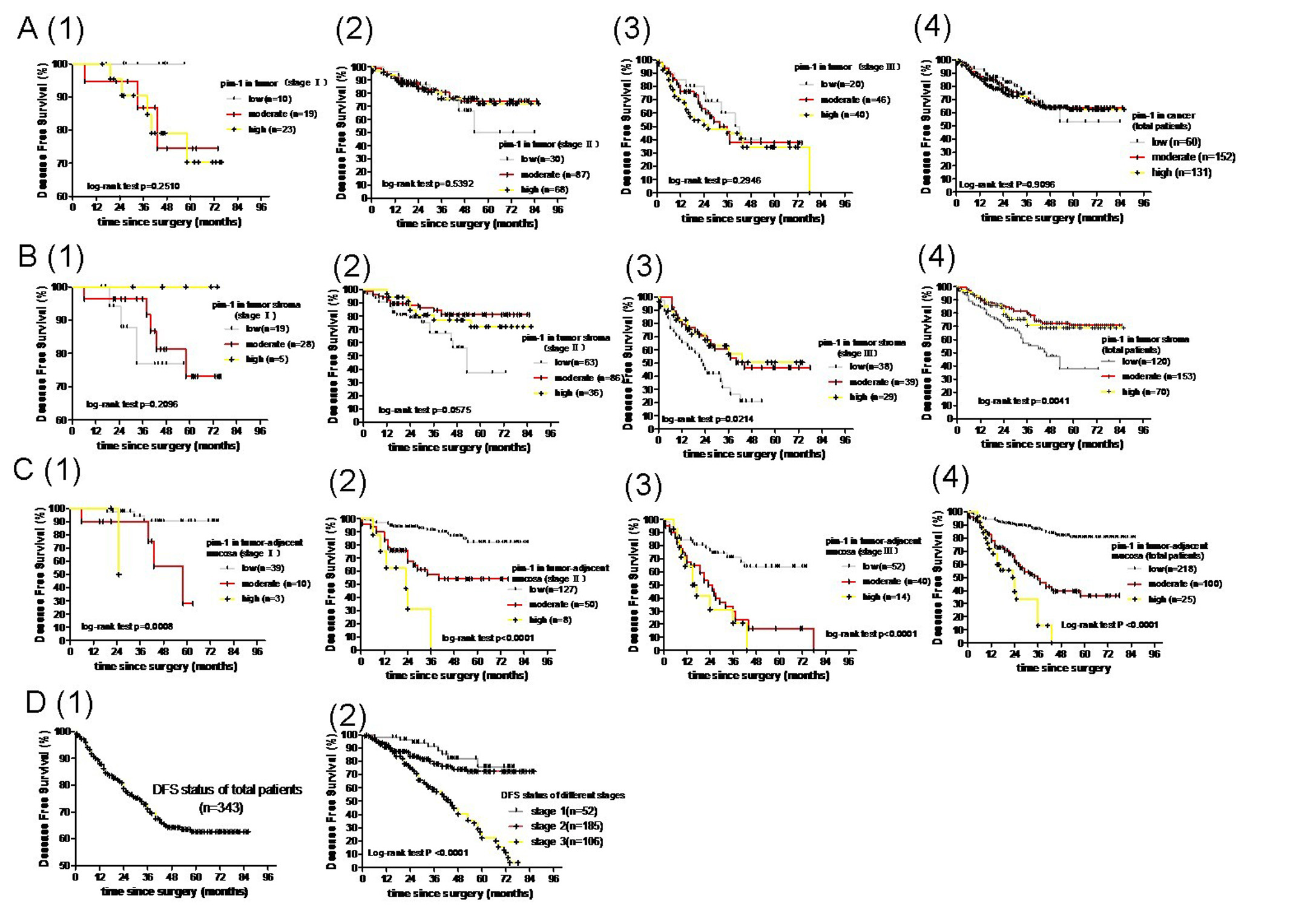

Supplement: Figure S2 — DFS curves generated by Kaplan–Meier analyses. (A) DFS curves of different pim-1 expression level in tumor of different group patients: 1, stage Ⅰ; 2, stageⅡ; 3, stage Ⅲ; 4, total patients. (B) DFS curves of different pim-1 expression level in tumor stroma of different group patients: 1, stage Ⅰ; 2, stageⅡ; 3, stage Ⅲ; 4, total patients. (C) DFS curves of different pim-1 expression level in tumor-adjacent mucosa of different group patients: 1, stage Ⅰ; 2, stageⅡ; 3, stage Ⅲ; 4, total patients. (D) DFS curves of total patients (1) and patients with stage Ⅰ, Ⅱ, Ⅲ diseases (2). (TIF) [file pone.0076693.s002.tif]

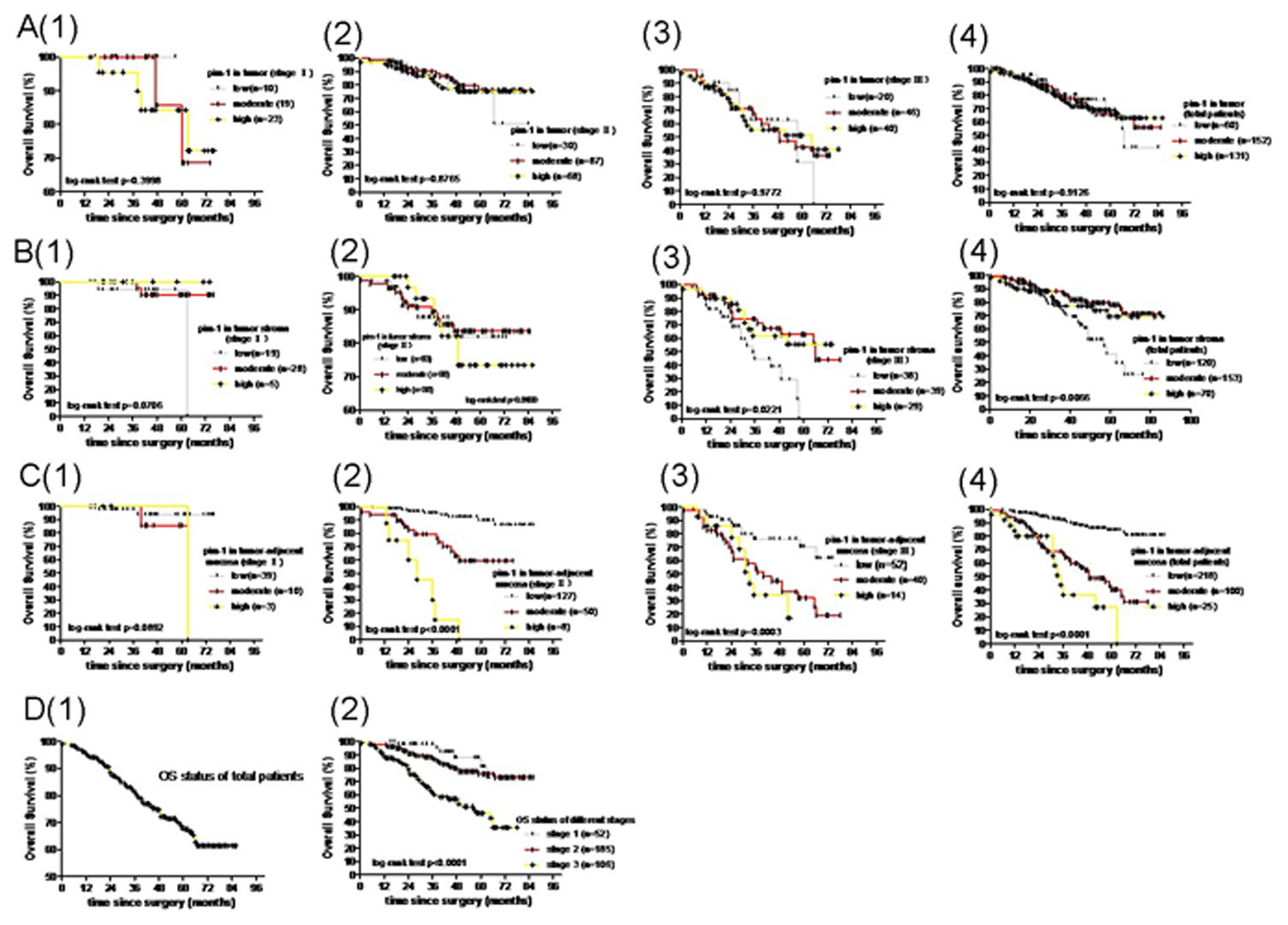

Supplement: Figure S3 — OS curves generated by Kaplan–Meier analyses. (A) OS curves of different pim-1 expression level in tumor of different group patients: 1, stage Ⅰ; 2, stageⅡ; 3, stage Ⅲ; 4, total patients. (B) OS curves of different pim-1 expression level in tumor stroma of different group patients: 1, stage Ⅰ; 2, stageⅡ; 3, stage Ⅲ; 4, total patients. (C) OS curves of different pim-1 expression level in tumor-adjacent mucosa of different group patients: 1, stage Ⅰ; 2, stageⅡ; 3, stage Ⅲ; 4, total patients. (D) OS curves of total patients (1) and patients with stage Ⅰ, Ⅱ, Ⅲ diseases (2). (TIF) [file pone.0076693.s003.tif]
