# Supplementary material for: Expression of pim-1 in Tumors, Tumor Stroma and Tumor-Adjacent Mucosa Co-Determines the Prognosis of Colon Cancer Patients
Source: PLoS One. 2013 Oct 7;8(10):e76693. doi: 10.1371/journal.pone.0076693 (PMC3792018; doi:10.1371/journal.pone.0076693)
Supplement: Table S1 — Clinicopathological correlation of pim-1 expression in colon cancer. (DOC) [file pone.0076693.s004.doc]

**Table S1.** Clinicopathological correlation of pim-1 expression in colon cancer.

| Variables | Pim-1 expression in tumor-adjacent mucosa (%) Pim-1 expression in tumor stroma (%) | | | | | | | | |
| --- | --- | --- | --- | --- | --- | --- | --- | --- | --- |
|  | low | moderate | high | *P ^※^* |  | Low | moderate | high | *p^※^* |
| **Age (years)** |  | | | 0.8833 |  |  | | | 0.2307 |
| <60 y | 62.42  (103/165) | 29.70  (49/165) | 7.79  (13/165) |  |  | 39.39  (65/165) | 40.61  (67/165) | 20.00  (33/165) |  |
| ≥60 y | 64.61  (115/178) | 28.65  (51/178) | 6.74  (12/178) |  |  | 30.90  (55/178) | 48.31  (86/178) | 25.00  (37/178) |  |
| **Gender** |  | | | 0.3457 |  |  | | | 0.2696 |
| Male | 66.84  (131/196) | 26.53  (52/196) | 6.63  (13/196) |  |  | 31.63  (62/196) | 47.96  (94/196) | 20.41  (40/196) |  |
| Female | 59.18  (87/147) | 32.65  (48/147) | 8.16  (12/147) |  |  | 39.46  (58/147) | 40.14  (59/147) | 20.41  (30/147) |  |
| **WHO grade** |  | | | 0.2265 |  |  | | | 0.9674 |
| G1 | 67.35  (33/49) | 24.49  (12/49) | 8.16  (4/49) |  |  | 36.73  (18/49) | 44.90  (22/49) | 18.37  (9/49) |  |
| G2 | 65.13  (170/261) | 28.35  (74/261) | 6.51  (17/261) |  |  | 34.10  (89/261) | 44.83  (117/261) | 21.07  (55/261) |  |
| G3 | 45.45  (15/33) | 42.42  (14/33) | 12.12  (4/33) |  |  | 39.39  (13/33) | 42.42  (14/33) | 18.18  (6/33) |  |
| **T status** |  | | | 0.2119 |  |  | | | 0.5680 |
| T 0/1 | 66.67  (4/6) | 33.33  (2/6) | 0  (0/6) |  |  | 16.67  (1/6) | 66,67  (4/6) | 16.67  (1/6) |  |
| T2 | 71.70  (38/53) | 18.87  (10/53) | 9.43  (5/53) |  |  | 39.62  (21/53) | 49.06  (26/53) | 11.32  (6/53) |  |
| T3 | 64.58  (155/240) | 28.75  (69/240) | 6.67  (16/240) |  |  | 34.58  (83/240) | 43.75  (105/240) | 21.67  (52/240) |  |
| T4 | 47.72  (21/44) | 43.18  (19/44) | 9.09  (4/44) |  |  | 34.09  (15/44) | 40.91  (18/44) | 25.00  (11/44) |  |
| **N status** |  | | | 0.0004 |  |  | | | 0.0347 |
| N0 | 70.04  (166/237) | 25.32  (60/237) | 4.64  (11/237) |  |  | 34.60  (82/237) | 48.10  (114/237) | 17.30  (41/237) |  |
| N1 | 53.23  (33/62) | 38.71  (24/62) | 8.06  (5/62) |  |  | 30.65  (19/62) | 40.32  (25/62) | 29.03  (18/62) |  |
| N2 | 43.48  (10/23) | 30.43  (7/23) | 26.09  (6/23) |  |  | 39.13  (9/23) | 21.74  (5/23) | 39.13  (9/23) |  |
| N3 | 42.86  (9/21) | 42.86  (9/21) | 14.29  (3/21) |  |  | 47.62  (10/21) | 42.86  (9/21) | 9.52  (2/21) |  |
| **TNM stages** |  | | | 0.0015 |  |  | | | 0.0826 |
| Stage Ⅰ | 75.00  (39/52) | 19.23  (10/52) | 5.77  (3/52) |  |  | 36.54  (19/52) | 53.85  (28/52) | 9.62  (5/52) |  |
| Stage Ⅱ | 68.65  (127/185) | 27.03  (50/185) | 4.32  (8/185) |  |  | 34.05  (63/185) | 46.49  (86/185) | 19.46  (36/185) |  |
| Stage Ⅲ | 49.06  (52/106) | 37.74  (40/106) | 13.21  (14/106) |  |  | 35.85  (38/106) | 36.79  (39/106) | 27.36  (29/106) |  |

^※^Chi-square test. Abbreviation: T, tumor; N, node.
