# Supplementary material for: Expression of pim-1 in Tumors, Tumor Stroma and Tumor-Adjacent Mucosa Co-Determines the Prognosis of Colon Cancer Patients
Source: PLoS One. 2013 Oct 7;8(10):e76693. doi: 10.1371/journal.pone.0076693 (PMC3792018; doi:10.1371/journal.pone.0076693)
Supplement: Table S2 — Predictive variables for DFS and OS of patients with stage i disease by univariate survival analysis. (※log-rank test.). (DOC) [file pone.0076693.s005.doc]

**Table S2.** Predictive variables for DFS and OS of patients with stage Ⅰ disease by univariate survival analysis. (^※^log-rank test. )

| Variables | P  cases | DFS | | OS | |
| --- | --- | --- | --- | --- | --- |
|  |  | 5 years’ survival, % | p^※^ | 5 years survival, % | p^※^ |
| **Pim-1 (tumors)** | 52 |  | 0.2510 |  | 0.3998 |
| low | 10 | 100.00 |  | 100.00 |  |
| moderate | 19 | 74.44 |  | 85.71 |  |
| high | 23 | 70.34 |  | 84.23 |  |
| **pim-1(tumor-adjacent mucosa)** | 52 |  | 0.0008 |  | 0.0915 |
| low | 39 | 90.31 |  | 93.69 |  |
| moderate | 10 | 28.13 |  | 85.71 |  |
| high | 3 | 0 |  | 0 |  |
| **pim-1 (tumor stroma)** | 52 |  | 0.2096 |  | 0.1975 |
| low | 19 | 76.83 |  | 94.12 |  |
| moderate | 28 | 73.23 |  | 90.48 |  |
| high | 5 | 100.00 |  | 100.00 |  |
| **PTS** | 52 |  | 0.0075 |  | 0.0384 |
| low | 14 | 100.00 |  | 100.00 |  |
| moderate | 29 | 78.97 |  | 84.48 |  |
| high | 9 | 0 |  | 75.00 |  |
