# Supplementary material for: Expression of pim-1 in Tumors, Tumor Stroma and Tumor-Adjacent Mucosa Co-Determines the Prognosis of Colon Cancer Patients
Source: PLoS One. 2013 Oct 7;8(10):e76693. doi: 10.1371/journal.pone.0076693 (PMC3792018; doi:10.1371/journal.pone.0076693)
Supplement: Table S3 — Predictive variables for DFS and OS of patients with stage ii disease by univariate survival analysis. (DOC) [file pone.0076693.s006.doc]

**Table S3.** Predictive variables for DFS and OS of patients with stage Ⅱ disease by univariate survival analysis.

| Variables | P  cases | DFS | | OS | |
| --- | --- | --- | --- | --- | --- |
|  |  | 5 years’ survival, % | p^※^ | 5 years survival, % | p^※^ |
| **Pim-1 (tumors)** | 185 |  | 0.6388 |  | 0.8765 |
| low | 30 | 50.00 |  | 76.55 |  |
| moderate | 87 | 74.00 |  | 76.08 |  |
| high | 68 | 72.23 |  | 75.23 |  |
| **pim-1 (tumor-adjacent mucosa)** | 185 |  | <0.0001 |  | <0.0001 |
| low | 127 | 81.96 |  | 89.51 |  |
| moderate | 50 | 54.27 |  | 59.51 |  |
| high | 8 | 0 |  | 0 |  |
| **pim-1 (tumor stroma)** | 185 |  | 0.0575 |  | 0.8109 |
| low | 63 | 37.45 |  | 81.81 |  |
| moderate | 86 | 81.20 |  | 83.68 |  |
| high | 36 | 72.05 |  | 73.49 |  |
| **PTS** | 185 |  | <0.0001 |  | <0.0001 |
| low | 63 | 92.46 |  | 97.56 |  |
| moderate | 89 | 67.71 |  | 71.36 |  |
| high | 33 | 27.38 |  | 38.75 |  |

^※^log-rank test.
