# Supplementary material for: Expression of pim-1 in Tumors, Tumor Stroma and Tumor-Adjacent Mucosa Co-Determines the Prognosis of Colon Cancer Patients
Source: PLoS One. 2013 Oct 7;8(10):e76693. doi: 10.1371/journal.pone.0076693 (PMC3792018; doi:10.1371/journal.pone.0076693)
Supplement: Table S4 — Predictive variables for DFS and OS of patients with stage iii disease by univariate survival analysis. (DOC) [file pone.0076693.s007.doc]

**Table S4.** Predictive variables for DFS and OS of patients with stage Ⅲ disease by univariate survival analysis.

| Variables | P  cases | DFS | | OS | |
| --- | --- | --- | --- | --- | --- |
|  |  | 5 years’ survival,% | p^※^ | 5 years survival,% | p^※^ |
| **Pim-1 (tumors)** | 106 |  | 0.2946 |  | 0.9772 |
| low | 20 | <40.00 |  | 31.25 |  |
| moderate | 46 | 37.82 |  | 42.51 |  |
| high | 40 | 34.47 |  | 51.25 |  |
| **pim-1 (tumor-adjacent mucosa)** | 106 |  | <0.0001 |  | 0.0003 |
| low | 52 | 63.91 |  | 70.30 |  |
| moderate | 40 | 16.69 |  | 32.14 |  |
| high | 14 | 0 |  | 17.14 |  |
| **pim-1 (tumor stroma)** | 106 |  | 0.0214 |  | 0.0221 |
| low | 38 | 0 |  | 0 |  |
| moderate | 39 | 46.55 |  | 62.93 |  |
| high | 29 | 50.45 |  | 55.47 |  |
| **PTS** | 106 |  | <0.0001 |  | <0.0001 |
| low | 23 | 91.67 |  | 100.00 |  |
| moderate | 51 | 33.19 |  | 41.73 |  |
| high | 32 | 0 |  | 17.22 |  |

^※^log-rank test.
