# Supplementary material for: Expression of pim-1 in Tumors, Tumor Stroma and Tumor-Adjacent Mucosa Co-Determines the Prognosis of Colon Cancer Patients
Source: PLoS One. 2013 Oct 7;8(10):e76693. doi: 10.1371/journal.pone.0076693 (PMC3792018; doi:10.1371/journal.pone.0076693)
Supplement: Table S5 — Pim-1 expression in tumors, tumor-adjacent mucosa and tumor stroma. (DOC) [file pone.0076693.s008.doc]

**Table S5.** Pim-1 expression in tumors, tumor-adjacent mucosa and tumor stroma.

| Pim-1 expression | Low | Moderate | High | P value |
| --- | --- | --- | --- | --- |
| Tumor (n) | 60 | 152 | 131 | 0.0000 |
| tumor stroma (n) | 120 | 153 | 70 |
| tumor-adjacent mucosa (n) | 218 | 100 | 25 |  |
